# Supplementary material for: Comparative Tensile Properties and Collagen Patterns in Domestic Cat (Felis catus) and Dog (Canis lupus familiaris) Ovarian Cortical Tissues
Source: Bioengineering (Basel). 2023 Nov 4;10(11):1285. doi: 10.3390/bioengineering10111285 (PMC10669533; doi:10.3390/bioengineering10111285)
Supplement: Supplementary file 1 [file bioengineering-10-01285-s001.zip › bioengineering-2667059-supplementary.pdf]

Supplemental Table S1. Details of the animals used in the study, with 'na' indicating where data are not available.

Species	Age (yr)	Breed	Age Category	Note
Cat	0.5	-	Adult	
	0.5	-	Adult	
	0.58	-	Adult	
	1	-	Adult	in heat
	1.5	-	Adult	
	2	-	Adult	
	na	-	Adult	in heat
	0.15	-	Prepubertal	MMP study
	0.15	-	Prepubertal	MMP study
	0.15	-	Prepubertal	MMP study
	0.19	-	Prepubertal	MMP study
	0.25	-	Prepubertal	
	0.25	-	Prepubertal	MMP study
	0.33	-	Prepubertal	
	0.33	-	Prepubertal	MMP study
	0.42	-	Prepubertal	
	0.5	-	Prepubertal	
	na	-	Prepubertal	
Dog	0.92	na	Adult	
	1	Terrier	Adult	
	1	Pitbull	Adult	
	1	Shih tzu	Adult	
	2	Labrador	Adult	
	2	Pitbull	Adult	
	3	Chihuahua	Adult	
	na	German Shepherd	Adult	
	0.33	Terrier X	Prepubertal	
	0.42	na	Prepubertal	MMP study
	0.42	Boston Terrier	Prepubertal	MMP study
	0.42	Boston Terrier	Prepubertal	MMP study
	0.50	Maltese	Prepubertal	
	0.50	na	Prepubertal	
	0.50	Boston Terrier	Prepubertal	
	0.50	Jack Russel Terrier	Prepubertal	
	0.58	Blue Tic	Prepubertal	
	0.58	Border Collie	Prepubertal	
	0.58	Terrier	Prepubertal	MMP study
	0.58	Pitbull	Prepubertal	MMP study
	0.67	Boxer	Prepubertal	
	0.83	na	Prepubertal	MMP study
	0.83	Labrador	Prepubertal	MMP study
	na	Chihuahua	Prepubertal	
	na	Hound	Prepubertal	

Supplemental Figures

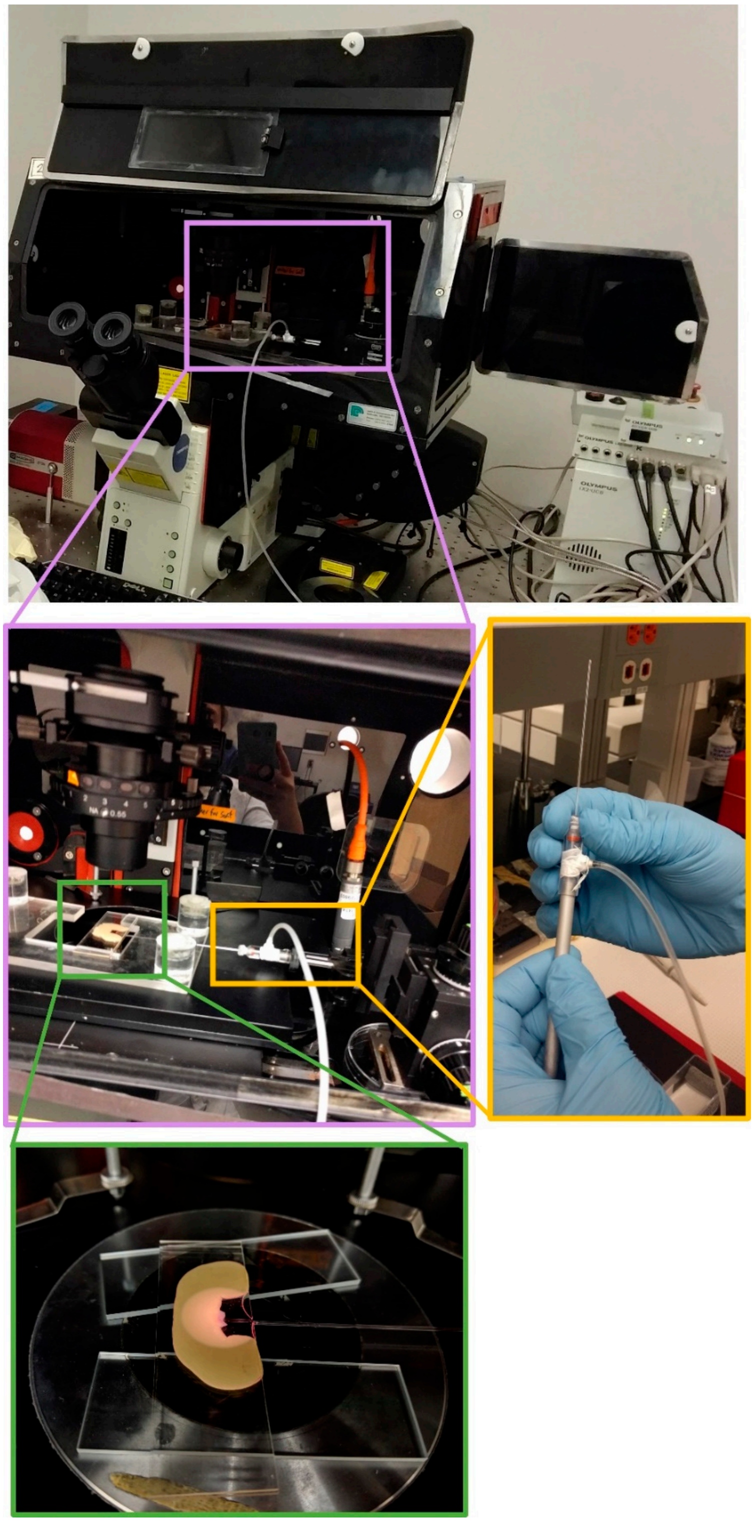

Supplemental Figure S1. Micropipette aspiration setup for tensile testing, with inverted microscope fitted with on-stage chamber to minimize air/light contamination during image

capture, and anti-vibration table. Experiment setup (purple box) included tissue sample sandwiched between glass slides (green box) with glass capillary attached to pneumatic transducer introduced via micromanipulation apparatus between the slides to contact the tissue.

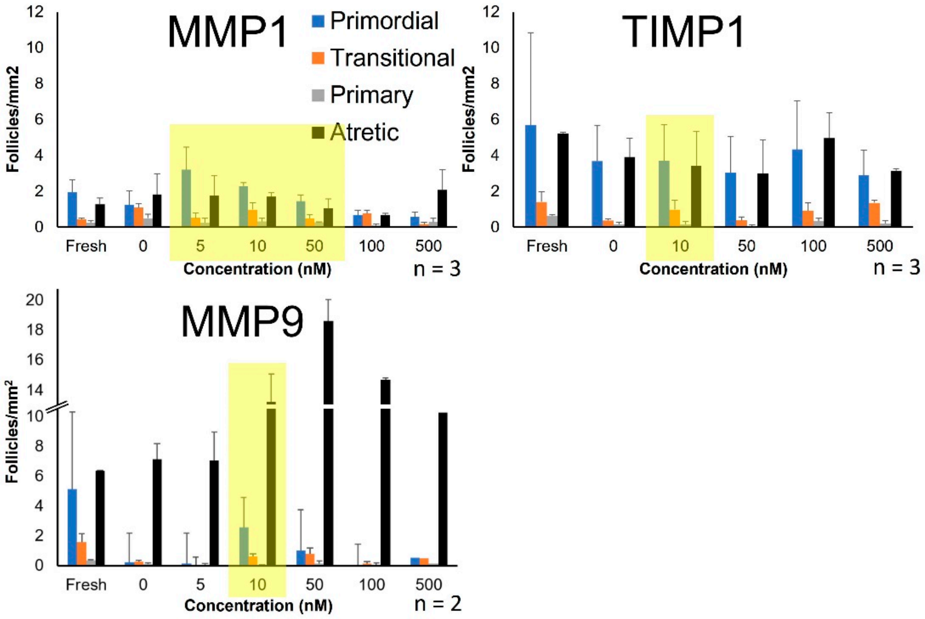

Supplemental Figure S2. Density of primordial, transitional, primary, and atretic follicles in domestic cat ovarian tissue after 14-day culture in various concentrations (0, 10, 50, 100, and 500 ng/ml) of MMP1, TIMP1, and MMP9. Yellow highlights indicate concentration regions wherein high proportions of primordial and transitional stage follicles were maintained, while minimizing proportions of atretic follicles for toxicological assessment of MMP and TIMPs.

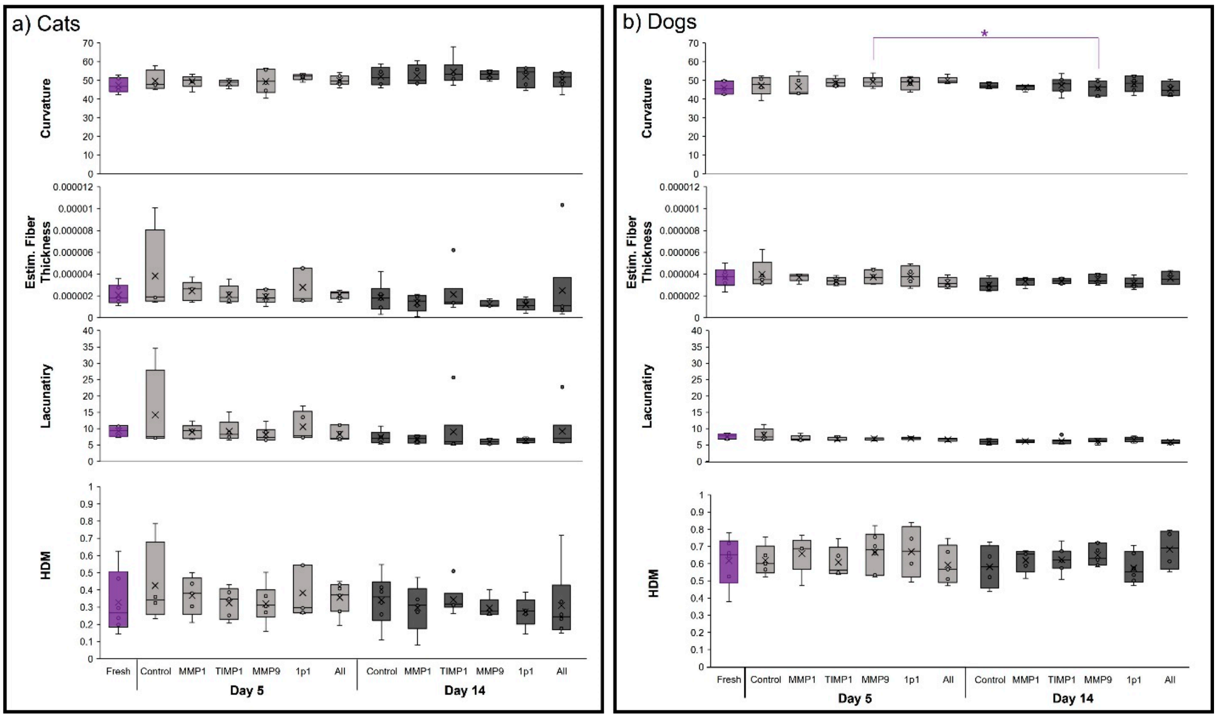

Supplemental Figure S3. Additional TWOMBLI results for a) cats (n = 6) and b) dogs (n = 7) analyzed for collagen patterns, including curvature, estimated fiber thickness, lacunarity, and high-density matrix (HDM). Asterisks (*) denote differences between treatments, with $P < 0.05$ via non-parametric Wilcoxon test.
